# Revelation of subclinical left ventricular diastolic dysfunction in patients with type 2 diabetes mellitus using 2016 ASE/ EACVI guidelines

**DOI:** 10.22088/cjim.12.4.586

**Published:** 2021

**Authors:** Srivatsa Raghothama, Akshay Rao

**Affiliations:** 1 Department of General Medicine, Justice K.S Hegde Medical Academy, Mangalore, Karnataka, India; 2 Department of General Medicine, Ramaiah Medical College, Bangalore, Karnataka, India

**Keywords:** Left ventricular diastolic dysfunction, Diabetic cardiomyopathy, Diastology, Tissue Doppler imaging, 2016 ASE/EACVI guidelines

## Abstract

**Background:**

Few studies have used the 2016 American Society of Echocardiography/ European Association of Cardiovascular Imaging (ASE/EACVI) guidelines to detect left ventricular diastolic dysfunction (LVDD) among asymptomatic normotensive type 2 diabetes mellitus (T2DM) patients.

**Methods:**

200 asymptomatic non-hypertensive diabetic cases and 281 controls matched for age and body mass index without evidence of arrhythmias, valvular, myocardial, pericardial or coronary artery disease underwent diastology assessment using 2 dimensional and M-mode echocardiography along with tissue Doppler imaging.

**Results:**

The presence of LVDD was seen to be significantly higher among the members of the diabetic group compared to the controls (35 vs. 14; P=0.001). The diabetics with LVDD had a longer duration of diabetes {8.04±7.75 vs. 5.27±5.49 years; P=0.04}, along with higher glycated hemoglobin (HbA1c) {8.40±1.38 vs. 7.80±1.60% ; P=0.05}, fasting blood glucose (FBS) {211.35±78.15 vs. 187.89±107.90 mg/dL; P=0.009, 2 hour post prandial blood glucose} (PPBS) {237.89±107.9 vs. 211.35±78.15 mg/dL; P=0.04}, serum triglyceride (TG) {246.91±171.82 vs. 163.44±99.37 mg/dL; P=0.008} yet had lower serum very low density lipoprotein levels (VLDL) {19.74±15.01 vs. 27.61±17.89 mg/dL; P=0.01}.

**Conclusion:**

This is one of the few studies so far to have demonstrated a higher occurrence of LVDD specifically among asymptomatic normotensive T2DM patients using the 2016 ASE/EACVI guidelines.

The heart is one of the organs affected in patients who suffer from type 2 diabetes mellitus (T2DM). In 2013, the American College of Cardiology Foundation, the American Heart Association, and the European Society of Cardiology in collaboration with the European Association for the Study of Diabetes, recognized that ‘diabetic cardiomyopathy’ is a syndrome characterized by the impairment in ventricular function among patients with T2DM without coronary artery disease (CAD) and systemic hypertension (1). Despite this, the existence of ‘diabetic cardiomyopathy’ as a distinct entity has been doubted due to the frequent association of T2DM with multiple confounders which may lead to cardiac dysfunction, such as advanced age and coexisting cardiovascular risk factors (2, 3). The cardiac involvement in T2DM may occur via several mechanisms including the induction of atherosclerotic CAD, inappropriate upregulation of the neurohormonal axis (sympathetic drive & renin angiotensin aldosterone), oxygen free radicle generation, disruption in the modulation of the immune system, enhanced insulin resistance as well as dysfunction of the coronary endothelium. T2DM also frequently coexists with other disorders that lead to cardiac disease such as systemic hypertension, hyperlipidemia, and obesity (4).

The cardia may be affected in the early stages of T2DM, initially producing an asymptomatic left ventricular diastolic dysfunction (LVDD) and may subsequently progress to the stage of heart failure with preserved ejection fraction (HFpEF). This pre-clinical LVDD may represent a reversible stage of cardiac damage. Indeed in the empagliflozin, cardiovascular outcome and mortality in type 2 DM (EMPA-REG OUTCOME) trial, it was demonstrated that in comparison to placebo, administration of empagliflozin produced early improvement in cardiac outcomes (1, 4, 5). Since there is paucity of evidence directed treatment options in HFpEF, there is a need to screen patients with T2DM early in the pre-clinical stage. The classic 2 dimensional (2D) echocardiography and Doppler parameters have had limitations in diagnosing LVDD and as such the criteria for its diagnosis have been revised several times. Tissue Doppler imaging (TDI) is a recent echocardiographic approach that has allowed the measurement of the longitudinal motion velocity of the mitral annulus and may be vital in diagnosing asymptomatic LVDD among patients with T2DM (5). In light of this, in 2016, the American Society of Echocardiography (ASE) and the European Association of Cardiovascular Imaging (EACVI) brought out new guidelines to define and grade diastolic dysfunction with greater specificity than the earlier guidelines (6). We decided to apply these guidelines to detect LVDD among normotensive T2DM patients without clinically overt heart failure. 

## Methods

**Study design**: Single center cross-sectional observational study 

**Study period**: Participants were enrolled between June 2016 and December 2019. 

**Place of the study**: The study was carried out in the Out Patient Department (OPD) and at the voluntary master health check-up (MHC) section of the Department of Internal Medicine at Ramaiah Medical College Hospitals in Bangalore, Karnataka in India. Institutional scientific and ethical review committee clearance was attained before the commencement of the study (Ethics code number MSRMC/EC/2016).

**Inclusion criteria**: All adults aged 18 years and above, attending the Medicine OPD or MHC were screened for inclusion into the study after duly taking informed consent. 

**Measurements**: Detailed history and physical examination findings including height, weight, total body surface area (BSA), body mass index (BMI) and blood pressure (BP) measurements were documented for all study participants. All participants underwent testing for fasting (FBS) and 2 hour post-prandial (PPBS) blood glucose levels (hexokinase method), glycated hemoglobin (HbA1c – high performance liquid chromatography method), fasting serum lipid parameters including total cholesterol, triglycerides (TG), high density lipoproteins (HDL), low density lipoproteins (LDL) and very low density lipoproteins (VLDL). The thyroid profile, complete blood counts, serum creatinine, standard 12 lead electrocardiogram (ECG), treadmill stress test (TMT) and spirometric pulmonary function testing was performed for all the participants.

A resting trans-thoracic 2D and motion mode (M mode) echocardiogram (ECHO) was performed for all participants with the application of TDI to assess for LVDD. The technician who performed the ECHO was kept unaware about this study. ECHO was conducted using the harmonic imaging mode by Philips Hd-15 machine (5-2 MHz multi-frequency probe). Trans-mitral flow velocities in the Apical four chamber view were recorded using pulsed wave Doppler (PWD) by placing the sample volume at the tip of the mitral leaflet. The readings from five consecutive cardiac cycles were averaged and used for the final analysis. Measurements included the trans-mitral peak modal velocity at early diastole (E in cm/s), peak modal velocity at late diastole (A in cm/s) and E/A ratio. On applying pulsed-wave TDI, average e’ velocity (peak modal velocity in early diastole at the leading edge of spectral waveform) was computed by placing the cursor at lateral (lateral e’ in cm/sec) and septal basal (septal e’ in cm/sec) regions by placing the sample volume at mitral valve leaflets. The left atrial maximum volume was calculated in the apical four and two chamber views freezing 1-2 frames before the opening of the mitral valve. This was then indexed to the total body surface area to obtain the left atrial volume index (LAVI in ml/m^2^). Peak tricuspid regurgitation jet velocity (TRV in m/sec) was calculated using continuous wave Doppler and colour flow imaging. Ejection fraction (EF) was obtained by using the modified Quinones formula. 

**Exclusion criteria: **Present or past cardiac symptoms like dyspnea or angina, hypertension (clinic BP >140/90 mmHg) or on pharmacotherapy for it, participants with type 1 diabetes mellitus, anemia, history of smoking, significant alcohol intake history, pregnancy and thyroid disease. Patients with clinical features of heart failure or EF <50% on echocardiography, those with known history or those newly found to have evidence of valvular, myocardial or pericardial disease, arrhythmias or/and CAD as well as presence of any structural heart diseases detected by history, physical examination, ECG, abnormal treadmill stress test or echocardiography. Those with only two of the four ASE/EACVI 2016 criteria for LVDD being fulfilled were deemed to be indeterminate for LVDD status and excluded. 

All participants were then divided as either diabetic cases or non-diabetic controls. All patients with pre-existing as well as newly diagnosed T2DM were classified into the diabetic group. A diagnosis of T2DM was made if one of the following was satisfied:

- Fasting blood sugar (FBS) ≥ 126 mg/dL on 2 separate occasions
- Two hour post prandial blood sugar (PPBS) > 200 mg/dL on 2 separate occasions
- HbA1C ≥ 6.5%

The diagnosis of LVDD was made in accordance with the ASE/EACVI 2016 guidelines, if at least three of the following four criteria were fulfilled:

1) Septal e’ < 7 cm/sec, lateral e’ < 10 cm/sec, 2) Average E/e’ ratio > 14, 3) LAVI > 34 mL/m^2^, 4) Peak TRV > 2.8 m/sec

Those satisfying only two criteria were labelled as indeterminate. Additionally, the presence of LVDD was also assessed using the 2009 ASE/ EAE guidelines (7). 

**Statistical Analysis:** Statistical Package for the Social Sciences (SPSS) 20 software was used for data analysis. The mean ± standard deviation (SD) for continuous variables and percentage for categorical variables were calculated respectively. Independent sample t-test and Pearson’s chi-square test was used to test for significance, for continuous and categorical variables, respectively. A p-value ≤ 0.05 was deemed to be significant.

## Results

After eliminating those with one or more of the exclusion criteria, a total of 523 participants underwent diastology assessment using the 2D ECHO and TDI. 17 diabetics and 25 non diabetics who were found to fulfill exactly two of the four ASE/EACVI criteria and thus excluded for having indeterminate LVDD status. A total of 481 participants were confirmed for the final analysis and were distributed into two groups, namely 200 diabetics and 281 healthy controls. There were no significant differences between the two groups with respect to their age {56.52 years (±9.94) vs. 55.50 years (±9.78); p=0.26} or BMI {27.40 Kg/m2 (±3.66) vs. 27.20 Kg/m2 (±4.45); P=0.86}, though there were significantly more number of female participants in the control group than in the diabetes group {66 (33.33%) vs 125 (45%); P=0.01} (table 1). 

It was found that according to the 2016 ASE/EACVI guidelines, LVDD was seen to be present in 35 of the diabetic participants and in 14 of the controls (P= 0.001). Further, on analyzing separately, the occurrence of LVDD was seen to be greater in both the female (9 vs. 7; P=0.05) and male (26 vs. 7; P=0.01) participants of the diabetic group (tables 1 & 2b). The comparison of LVDD rates according to the 2009 guidelines also revealed a higher occurrence of LVDD among diabetics (56 vs. 31, p=0.001). The echocardiographic parameters of all participants revealed that despite no significant difference in their EF, the indicators of diastolic function were significantly abnormal in the diabetic group in comparison with the controls (tables 2a and 2b).

**Table 1:** Attributes of the study participants

**Parameters**	**Diabetics (n=200)** **Total numbers (%) or** **Mean ± SD **	**Controls (n=281)** **Total numbers (%) or** **Mean ± SD**	**p value**
Age (years)	56.52 (±9.94)	55.50 (±9.78)	0.26
BMI (Kg/m^2^)	27.40 (±3.66)	27.20 (±4.45)	0.86
LVDD 2016 guidelines	35 (17.5%)	14 (4.98%)	0.001
Number of females in the group	66 (33.33%)	125 (45%)	0.01
Females with LVDD	9 (13.64%)	7 (5.60%)	0.05
Number of males in the group	134 (67%)	156 (55%)	0.03
Males with LVDD	26 (20.3%)	7 (4.50%)	0.01
LVDD 2009 guidelines	56 (28%)	31 (11%)	0.001

**Values are given as percentage or mean ± SD, SD = standard deviation, BMI = body mass index, LVDD = left ventricular diastolic dysfunction**

**Table 2 a:** Comparison of the Echocardiographic parameters

**Parameter **	**Diabetic (n=200)**	**Controls (n=281) **	**P**
	**Mean ± SD**	**Mean ± SD**	
E (m/s)	77.19 ±11.04	81.59 ± 9.79	0.001
A (m/s)	90.03 ± 21.01	83.56 ± 17.34	0.001
E/A	0.96 ± 8.71	1.28 ± 4.69	0.027
EF (%)	60.79 ± 5.34	60.97 ± 5.49	0.71
E/e’	9.17± 2.27	8.80 ± 1.81	0.05
Lateral e’ (m/s)	9.79 ± 2.27	10.62 ± 2.24	0.001
Septal e’ (m/s)	7.50 ± 1.99	8.27 ± 2.00	0.001
LAVI (ml/m^2^)	30.57 ± 11.04	26.67 ± 9.79	0.001
TR velocity (m/s)	2.08 ± 0.71	1.63 ± 0.55	0.001
DT (msec)	138.77± 27.57	145.28 ± 28.44	0.01
IVRT (msec)	91.70 ± 14.08	93.06 ± 14.52	0.81

**Values are given as Mean ± Standard Deviation (SD), E = trans-mitral peak modal velocity at early diastole, A = trans-mitral peak modal velocity at late diastole, EF = ejection fraction, e’ = peak early diastolic mitral valve annular velocity, LAVI = left atrial volume index, TR velocity = tricuspid regurgitant jet velocity, DT = deceleration time, IVRT = Isovolumetric relaxation time**

**Table 2 b:** Comparison of the Echocardiographic parameters based on gender

	**Males**		**p**	**Females**		**p**
	**Diabetic (n=134)**	**Control (n=156)**		**Diabetic (n=66)**	**Control (n=125)**	
	**Mean ± SD**			**Mean ± SD**		
E (m/s)	74.60 ± 17.21	80.05 ± 17.06	0.007	82.64 ±17.41	83.53 ±19.81	0.08
A (m/s)	83.54 ± 19.50	79.68 ± 15.76	0.043	1.03 ± 17.83	8.84 ±18.06	0.001
E/A	1.92 ± 8.05	1.54 ± 6.29	0.070	0.97 ±.29	1.79 ± 12.68	0.05
E/e’	11.63 ± 1.94	8.53 ± 1.60	0.041	10.53 ± 2.55	9.16 ± 1.99	0.01
Lateral e’ (m/s)	9.99 ± 2.18	10.59 ± 2.34	0.025	9.18 ± 2.41	10.67 ± 2.14	0.001
Septal e’ (m/s)	7.71 ± 1.84	8.46 ± 2.04	0.010	6.99 ± 2.19	8.04 ± 1.94	0.008
LAVI (ml/m^2^)	31.24 ± 10.70	26.6034 ± 9.84	0.07	27.90 ± 11.10	26.73 ± 9.78	0.06
TR velocity (m/s)	1.66 ± 0.59	1.81 ± 0.54	0.091	1.78 ± 0.41	1.58 ± 0.51	0.004

E = trans-mitral peak modal velocity at early diastole, A = trans-mitral peak modal velocity at late diastole, e’ = peak early diastolic mitral valve annular velocity, LAVI = left atrial volume index, TR velocity = tricuspid regurgitant jet velocity

A comparison of participants within the diabetic group showed that age and BMI did not differ among those with and without LVDD. Those with LVDD had a significantly higher FBS {187.89±107.90 mg/dL vs. 211.35±78.15 mg/dL; P=0.009}, PPBS {237.89±107.9 vs. 211.35±78.15 mg/dL; P=0.04} and HbA1c {8.40±1.38 % vs. 7.80±1.60%; P= 0.05}. The duration of diabetes was also observed to have been longer for those diabetics with LVDD {8.04±7.75 years vs. 5.27±5.49 years; P= 0.04}. The diabetics with LVDD had a significantly higher serum triglyceride level {246.91±171.82 mg/dL vs. 163.44±99.37 mg/dL; P=0.008} yet also had a lower VLDL level {19.74±15.01 mg/dL vs. 27.61±17.89 mg/dL; P=0.01}, but the rest of the lipid parameters did not differ significantly (table 3). 

**Table 3:** Comparison of diabetics with and without LVDD

**Parameter **	**Diabetics with LVDD (n=35)**	**Diabetics with no LVDD (n=165)**	**P**
Age (years)	58.14 ± 9.10	56.18 ± 10.10	0.26
BMI (Kg/m^2^)	29.50 ± 6.27	27.3 ± 3.90	0.32
Duration of DM (years)	8.04 ± 7.75	5.27 ± 5.49	0.04
HbA1c (%)	8.40 ± 1.38	7.80 ± 1.60	0.05
FBS (mg/dL)	211.35 ± 78.15	187.89 ± 107.90	0.009
PPBS (mg/dL)	237.89 ± 107.9	211.35 ± 78.15	0.04
Total cholesterol (mg/dL)	190.82 ± 68.71	177.50 ± 44.99	0.27
Triglycerides (mg/dL)	246.91 ± 171.82	163.44 ± 99.37	0.008
HDL (mg/dL)	36.60 ± 10.23	39.52 ± 10.24	0.13
LDL (mg/dL)	102.23 ± 34.81	106.25 ± 36.18	0.54
VLDL (mg/dL)	19.74 ± 15.01	27.61 ± 17.89	0.01

**LVDD = left ventricular diastolic dysfunction, BMI = body mass index, DM = diabetes mellitus, HbA1c= serum glycated haemoglobin, FBS = fasting blood sugar, PPBS = postprandial blood sugar, HDL = high density lipoproteins, LDL = low density lipoproteins, VLDL = very low density lipoproteins**

## Discussion

LVDD is thought to be central to the development of HFpEF (8). Several risk factors are said to contribute to the development of HFpEF including advancing age, hypertension, obesity, CAD apart from diabetes. The presence of these confounders have posed difficulties in carrying out studies on diabetic cardiomyopathy, some even question the existence of such an entity (2, 3). Diabetic cardiomyopathy is said to occur early in patients with T2DM characterized initially by LVDD, later followed by development of HFpEF, left ventricular hypertrophy (LVH) and systolic dysfunction (LVSD), and finally HF with reduced EF (HFrEF) (1). HFpEF has been shown to reduce the quality of life while increasing the rates of hospitalization and premature death. Large randomized clinical trials of therapies that have demonstrated improvement in outcomes of patients with HFrEF have failed to show similar benefit in patients with HFpEF (9-11). This underlines the need for early detection and conduct further studies to explore the underlying pathogenesis of LVDD.

The assessment of diastology has received a lot of importance in the recent years. The 2009 guidelines by ASE and European Association of Echocardiography (EAE) for LVDD were based on several echocardiographic parameters, but these were considered by many to be complicated. In 2016 the ASE/EACVI brought out new criteria for diagnosing LVDD in those with normal, as well as reduced EF, combining traditional 2D and M mode echocardiographic parameters with TDI parameters, thereby enhancing the specificity of these criteria, perhaps achieving this at the expense of some degree of the sensitivity (7). This guideline recommended new parameters, the number of criteria to be assessed to diagnose LVDD to include four parameters. Most studies on pre-clinical diabetic cardiomyopathy have thus far been conducted using the 2009 ASE/EAE criteria and have shown LVDD to be present in the absence of LVSD or LVH in a significant proportion of such patients. Through this study, we set out to determine if the presence of LVDD among asymptomatic normotensive diabetic cases was greater than in non-diabetic controls using the 2016 ASE/EACVI guidelines. 

We attempted to minimize the impact of confounding factors of LVDD either by having them excluded (hypertension, CAD, arrhythmia, structural heart disease, anemia and hypothyroidism) or by matching (age and BMI). The controls enrolled in the study were more in number than the cases. LVDD was seen to be higher in those with T2DM in our study as per the 2016 as well as the 2009 echocardiographic diagnostic guidelines. Though this is in agreement with previous studies, the proportion of diabetics with LVDD in our study was lower than previously reported (12-20), most likely because of the lower sensitivity of the newer guidelines but these differences need to be validated using cardiac catheterization which is the gold standard method of measuring intra-cardiac filling pressures (7, 21). The two groups were found to differ significantly with respect to the newer echocardiographic diastology parameters also (table 2). But past studies, including one that applied the 2016 guidelines, did not find LVDD to be more prevalent among asymptomatic normotensive diabetics (22). Such discrepancies have led to doubts over the very existence of diabetic cardiomyopathy as a separate entity.HFpEF is said to be more prevalent in women. In our study, though the number of women in the control group was more, the presence of LVDD was significantly higher in the women with T2DM. This was true for diabetic men, but the association was even stronger. The difference in the rates of LVDD among diabetic men and diabetic women was not found to be significant. Previous studies have shown a higher occurrence of LVDD in women with T2DM (5, 13, 16), while others have not found such female predilection (18).

To compare further, participants with LVDD in the diabetic group were seen to have a longer duration of T2DM, higher HbA1c, FBS and PPBS as against those diabetics without LVDD. This is in concurrence with previous diastology studies among diabetics that utilized the new and the older guidelines (5, 14, 15). One interesting observation though was the higher mean serum triglyceride levels and lower mean VLDL levels found in those diabetics in our study with LVDD. This has been described in previous studies, yet the relevance of this is uncertain. It is well known that hypertriglyceridemia frequently coexists with T2DM as a part of metabolic syndrome (13, 22, 23). It may only be highlighting the frequent accompaniment of comorbid factors with T2DM which may influence the development of LVDD.

A number of studies have been conducted in the past to detect LVDD in asymptomatic T2DM patients by employing the 2009 ASE/EAE guidelines. On reviewing the literature published online revealed that only few studies have been carried out in asymptomatic T2DM patients by applying the 2016 ASE/EACVI criteria to diagnose LVDD (5, 22). To the best of our knowledge, at the time of submission for publication, our research is only the third study in the world, and the first from India to have evaluated for the presence of LVDD specifically in normotensive T2DM patients without overt heart failure by employing the 2016 ASE/EACVI guidelines.

This study was carried out using a larger sample size compared to many previous studies. The ratio of the number of controls to cases was greater than 1. All practical attempts were made to reduce the effects of confounding factors. Limitations of our study include the cross sectional design and the fact that tests like coronary angiography were not used to exclude CAD, but subjecting asymptomatic patients to expensive and invasive tests in a resource-limited country like India would not have been appropriate.

In conclusions our study has illustrated that the occurrence of LVDD is more frequent among asymptomatic normotensive T2DM patients compared to healthy controls by applying the 2016 ASE/EACVI guidelines. This is a manifestation of diabetic cardiomyopathy which could be yet another complication of diabetes or a result of the interaction of multiple- associated comorbid conditions producing cardiac injury. In the study, a longer duration of T2DM, higher HbA1c, FBS, PPBS and TG as well as lower VLDL was seen to be significantly associated with the presence of LVDD among diabetics whereas there was no difference with respect to gender. There is a need to conduct larger prospective studies to confirm the association between LVDD with asymptomatic T2DM and to investigate the role of triglyceride levels in producing early LVDD. 
